# Effects of a Flavonoid-Rich Blackcurrant Beverage on Markers of the Gut-Brain Axis in Healthy Females: Secondary Findings From a 4-Week Randomized Crossover Control Trial

**DOI:** 10.1016/j.cdnut.2024.102158

**Published:** 2024-04-12

**Authors:** Nicola A Gillies, Brooke C Wilson, Jessica R Miller, Nicole C Roy, Andrew Scholey, Andrea J Braakhuis

**Affiliations:** 1 Discipline of Nutrition and Dietetics, University of Auckland, Auckland, New Zealand; 2 The Liggins Institute, University of Auckland, Auckland, New Zealand; 3 Department of Human Nutrition, University of Otago, Otago, New Zealand; 4 The Riddet Institute, Palmerston North, New Zealand; 5 The High-Value Nutrition National Science Challenge, New Zealand; 6 Nutrition Dietetics and Food, School of Clinical Sciences, Monash University, Melbourne, Australia; 7 Centre for Mental Health and Brain Sciences, Swinburne University, Melbourne, Victoria, Australia

**Keywords:** blackcurrant, cognition, mood, gut microbiome, flavonoid

## Abstract

The microbiota–gut–brain axis is a promising target to alleviate the growing burden of neurologic and mental health disorders. Dietary polyphenols act on multiple components of the microbiota–gut–brain axis, but this complex relationship requires further attention. This randomized, placebo-controlled, double-blind, crossover trial (ACTRN12622000850774) compared 4 wk of a commercially available flavonoid-rich blackcurrant beverage (FBB; 151 mg anthocyanins, 308 mg total polyphenols) with placebo in 40 healthy females (18–45 y). The primary outcome of stress reactivity was assessed by change in present feelings of stress, mood, and fatigue before and after completing a 20-min cognitive stressor [Purple multitasking framework (MTF)]. Secondary end points included cognitive performance (MTF), mood [profile of mood states (POMS)], sleep (Pittsburgh Sleep Quality Index), fecal microbiome composition and functional potential (shotgun sequencing), and blood biomarker concentrations (brain-derived neurotrophic factor, tryptophan, kynurenine, and interleukin 6). Statistical analyses were conducted on an intention-to-treat basis using linear mixed-effect models. Thirty-eight participants completed both intervention arms. There was no significant treatment effect on the primary outcome of stress reactivity. Compared with placebo, working memory (letter retrieval scores from MTF), and anxiety/tension and anger/hostility domains of the POMS improved with FBB supplementation (time × intervention interaction; *P* < 0.05). There were no treatment effects on gut microbiome composition or functional potential. Baseline abundances of *Bifidobacterium* genera and species (*Bifidobacterium longum* and *Bifidobacterium bifidum*) tended to be higher in participants with the greatest improvements in letter retrieval scores with FBB supplementation (nominally significant, *P* < 0.05)*.* In conclusion, 4-wk FBB supplementation improved secondary outcomes of working memory performance during multitasking and mood outcomes in healthy adult females. These results should be confirmed in a larger cohort with a longer duration of follow-up.

## Introduction

The microbiota–gut–brain axis is a potential target to curb the growing social and economic burden of mental health and neurologic disorders [[1], [2], [3]]. This dynamic network encompasses the bidirectional communication between the central nervous system and gastrointestinal tract, mediated by immune, neurotransmitter, neuroendocrine, and metabolic pathways alongside the trillions of bacteria comprising the resident gut microbiota [4].

Diverse aspects of diet can influence the microbiota–gut–brain axis, from whole diets to individual foods or bioactives, including polyphenols [5]. Polyphenols are a heterogenous group of bioactive compounds synthesized exclusively in plants, which share a common phenolic structure of hydroxyl groups on an aromatic ring [6]. The flavonoid class of polyphenols have been extensively researched, with their potential benefits for brain health attributed to antioxidant actions, neuroprotective effects, enhanced cerebrovascular blood flow, and stimulating neurogenesis (see Spencer et al. [7] for a comprehensive review). Flavonoids have more recently been recognized as prebiotic microbiota modulators, stimulating the proliferation of health-promoting species such as *Bifidobacterium* spp while inhibiting the growth of pathogenic species [8,9].

Despite the potential for flavonoids to enhance microbiota–gut–brain axis function through multiple pathways, their efficacy on cognition and mood varies greatly [[10], [11], [12]]. This may be related to low bioavailability [13,14], where an estimated 90%–95% of dietary polyphenols are not absorbed in the small intestine, reaching the colon where the gut microbiota transforms parent compounds into bioactive secondary metabolites [9,13]. The 2-way interplay between polyphenols and the gut microbiota, including prebiotic effects of polyphenols and biotransformation from the gut microbiota, is thought to underlie the ability of dietary polyphenols to produce their clinical effects or lack thereof [15,16]. This bidirectional relationship is highlighted in preclinical research, where specific gut microbiota communities were associated with the bioavailability and memory-enhancing effects of grape-derived polyphenol supplementation in a sleep-induced model of cognitive impairment, whereas clinical efficacy was attenuated by antibiotic-induced dysbiosis [17]. There have been remarkably few human intervention studies that have attempted to unravel this relationship [18,19], which theoretically could lead to targeted and enhanced intervention strategies.

Blackcurrants (*Ribes nigrum*) have received little research attention compared with other berry fruits yet are one of the richest and most variable sources of anthocyanins [15]. This study tested a commercially available blackcurrant-based beverage rich in anthocyanins (151 mg) and containing 150 mg *Pinus radiata* extract (pine bark, predominantly comprised of proanthocyanidin class of flavonoids) and 200 mg the amino acid L-theanine, here referred to as a flavonoid-rich blackcurrant beverage (FBB).

The objective of this research was to investigate the effects of 4-wk daily FBB supplementation compared with placebo in healthy female adults, on indicators of brain and mental health, biochemical parameters, and gut microbiome composition and predicted function. We also aimed to understand whether gut microbiome composition at baseline influenced cognitive or mood responses to the intervention or whether any changes in gut microbiome composition from baseline were associated with changes in behavioral parameters.

## Methods

### Study design

The trial followed a randomized, double-blinded, placebo-controlled, crossover study design. The LINK (polyphenol-rich drink for gut and brain health) study was conducted between July 2022 and January 2023 in Auckland, New Zealand, and was preregistered with the Australian New Zealand Clinical Trials Registry (ACTRN12622000850774). The protocol was developed in accordance with the Standard Protocol Items: Recommendations for Intervention Trials statement [20] and reported according to the CONSORT statement [21]. The procedures were approved by the Health and Disability Ethics Committee (2022 EXP 12513), and the study was conducted according to the guidelines laid down in the Declaration of Helsinki with all volunteers providing informed consent.

This study measured multiple outcomes related to the microbiome–gut–brain axis following 4 wk each of FBB and placebo beverages. Stress reactivity to a cognitive stressor [20-min Purple multitasking framework (MTF)] was selected as the primary outcome for this research, relating to both mental health and cognitive function [22,23]. Preregistered secondary outcomes include cognitive function measured by performance on the MTF, sleep measured by the Pittsburgh Sleep Quality Index (PSQI), mood measured by the profile of mood states questionnaire (POMS), biochemical parameters [serum tryptophan, kynurenine, IL-6, and brain-derived neurotrophic factor (BDNF)], and gut microbiome composition and functional potential. A crossover design was selected to reduce interindividual variation across these measures, particularly the significant variation observed with gut microbiome outcomes [24].

### Participants

Participants were recruited between July 2022 and September 2022 through opportunity sampling, including online advertising (social media and University of Auckland advertisements) and posters around the University of Auckland. Following expression of interest, potential participants completed an online screening questionnaire. The screening questionnaire included a modified version of the Diet Screening Tool (DST) to categorize participants into balanced optimal (*n* = 20) or suboptimal (*n* = 20) diet groups during recruitment, described in more detail further.

Eligible participants were healthy females aged 18–45 y, with a BMI between 18 and 30 kg/m^2^, who reported not using recreational/illicit drugs or to excessively consume alcohol (>15 standard drinks per week), and had access to the internet and technology required to complete online questionnaires. Participants had not used antibiotics in the 4 wk before beginning the intervention and agreed to abstain from taking probiotics, prebiotics, or supplements/herbal extracts that might reasonably be expected to interfere with mood or cognition for 4 wk before and for the duration of the study. Participants confirmed that they were not pregnant, nor intending to become pregnant during the trial. Based on self-report, participants had not been treated for anxiety, depression, or psychiatric disorders within the last 2 y and were free from neurologic (e.g., Parkinson disease), gastrointestinal (e.g., inflammatory bowel disease), and metabolic (e.g., cardiovascular disease) disorders or cognitive impairment. Participants were excluded if they used medications likely to interfere with normal digestive processes (e.g., laxatives) or if they reported a sensitivity to any ingredient in the treatments.

### Treatment

Participants received a 4-wk supply each of the 300-mL FBB and placebo beverage, separated by a 4-wk washout period. Beverages were supplied and blinded by Ārepa IP. Treatment order was determined using a computer-generated randomizer (www.randomizer.org) prepared by an independent researcher and managed centrally on REDCap during the trial. Sequences were stratified according to DST group, with a balance of optimal and suboptimal diets allocated to each treatment order. All research staff involved in the collection and analysis of data remained blinded until all aspects of the study were completed, including statistical analysis.

The FBB contains blackcurrant juice and extracts, 150 mg Enzogenol (*P. radiata* extract, primarily comprised of proanthocyanidins), and 200 mg L-theanine. The placebo beverage was a taste-matched and appearance-matched control, which was also matched for macronutrient and vitamin C (Table 1). The placebo beverage has successfully been used in a prior RCT investigating the same active intervention [25]. The phenolic content of the beverages was identified and quantified by an independent research laboratory (Cawthron Institute, New Zealand) using liquid chromatography methods coupled with photodiode array detection and mass spectrometry. The FBB provided 151 mg anthocyanins, with a total polyphenol content of 308 mg, whereas the placebo beverage contained a negligible polyphenol content of 22 mg (Table 1).

**TABLE 1:** Phenolic and nutritional content of treatment beverages.

	Active intervention beverage	Placebo control beverage
Total polyphenols (mg)	308	22
Anthocyanins (mg)	151	7
Energy (kJ)	155	155
Protein (g)	0.4	0.4
Fat total (g)	0	0
Fat saturated (g)	0	0
Carbohydrate (g)	23	23
Sugars (g)	14.8	14.8
Sodium (g)	8	8
Vitamin C (mg)	90	90

Phenolic content was independently analyzed by a research laboratory (Cawthron Institute) using liquid chromatography methods coupled with photodiode array detection and mass spectrometry.

The beverages were produced in a single batch in the week before the intervention starting, then stored in a dark, temperature-controlled room before distribution to participants. Participants were directed to keep the beverages in their fridge or a cool, dark place at home to minimize polyphenol degradation and asked to consume their 300-mL beverage daily at a similar time of day for 4 wk.

Participants were asked to maintain their normal diet and lifestyle habits for the duration of the study. Intervention compliance was assessed by weekly online questionnaires, as the return of bottles was considered impractical. Compliance questionnaires included the number of beverages the participant had consumed that week, the reason for not consuming all 7 allocated beverages (if applicable), time of day the beverages were consumed, and if they had made any changes to their diet or lifestyle over the last week. Compliance to the treatment was recorded as a cumulative score (percentage of beverages consumed out of the total 28 provided), with minimum compliance requirements defined a priori as 80%.

### Procedure

Participants were required to visit the Clinical Research Centre (University of Auckland, New Zealand) on 5 separate occasions. An enrollment/familiarization visit took place ∼2 wk before the first testing visit. Following written informed consent, participants provided health and demographic data and then completed the 2-min built-in familiarization session of the MTF to minimize learning effects. Participants were given a 3-d food record to complete before their first testing visit and given a fecal sample collection kit to collect a fecal sample in the 24 h before their first testing visit. Participants were randomly allocated to a treatment order at the end of the enrollment/familiarization visit.

**FIGURE 1:**
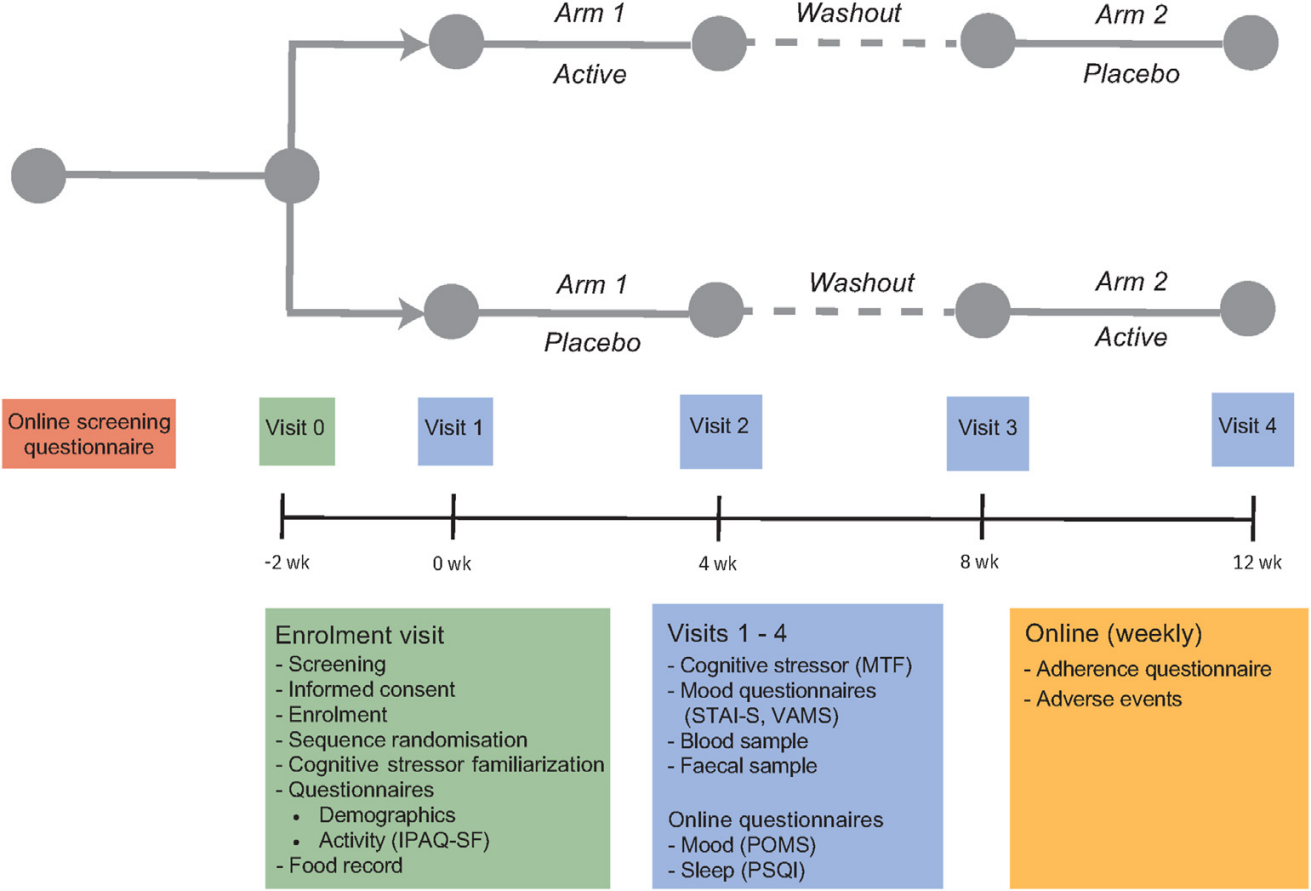
LINK study schematic. IPAQ-SF, International Physical Activity Questionnaire—short form; MTF, multitasking framework; POMS, profile of mood states; PSQI, Pittsburgh Sleep Quality Index; STAI-S, state trait anxiety inventory—state portion; VAMS, visual analog mood scale.

Participants were asked to abstain from caffeine for 12 h and alcohol for 24 h before each testing visit. Participants attended their testing visits between 07:00 and 10:00 following an overnight fast, with the time kept consistent across the 4 visits for each participant. Participants completed stress reactivity and cognitive measures before providing a fasted blood sample. In-person contact time was kept minimal owing to ongoing COVID-19 requirements at the time this study was conducted, and participants were instructed to complete online versions of the sleep and mood questionnaires within 24 h (Figure 1). Participants were provided with their 28 beverages at the end of visits 1 and 3 and were provided with another stool collection kit at the end of visits 1–3.

### Dietary assessment

#### Dietary screening tool

The Australian modification of the DST [26,27] was used, requiring only minimal adaptation to the New Zealand context (e.g., updating the name of popular fast food restaurants). The DST comprises 20 items, with 18 items related to the frequency of consumption of particular foods (e.g., “How often do you usually eat wholegrain breads or crackers?”) and 2 items related to the number of servings consumed (e.g., “How many different vegetable servings do you usually have at your main meal of the day?”). The DST has a maximum score of 104, with higher scores indicating better diet quality. Cutoff scores of ≤59 (suboptimal) and ≥60 (optimal) were used, which have been previously validated in middle-aged adults [28] and shown to discriminate nutrient intake, blood nutrient status, mood, and cognition in middle-aged adults [27]. The intent of a priori dietary screening was to capture a wider variety of dietary intakes than typically achieved with opportunity sampling in a healthy population, countering the issue of self-selection bias [29].

#### Food records

Participants completed a 3-d food record before their first testing visit. Instructions on how to complete the food record with as much detail as possible were provided by a registered dietitian at the enrollment/familiarization study visit, along with a standardized template to use. All records were checked for accuracy and completeness by a registered dietitian, with any concerns resolved in person. One student dietitian (JRM) entered the food records into FoodWorks software (version 10; Xyris), with 10% of the records randomly crosschecked by another student dietitian. The mean daily macronutrient and micronutrient composition of participant diets were calculated from the nutrient composition data primarily from the New Zealand FOOD files 2016 database or the AusBrands 2019 and AusFoods 2019 databases if a suitable item was not available in the New Zealand database. Energy overreporting and underreporting was checked from the 3-d food records, and all participants had plausible energy intakes (range: 5281–14,180 kJ/d). Nutrient intake was compared with age-specific and sex-specific estimated mean requirements or adequate intakes (if estimated mean requirements were unavailable) according to the joint Australia and New Zealand Nutrient Reference Values [30].

### Stress reactivity and cognitive measures

#### Purple MTF

**FIGURE 2:**
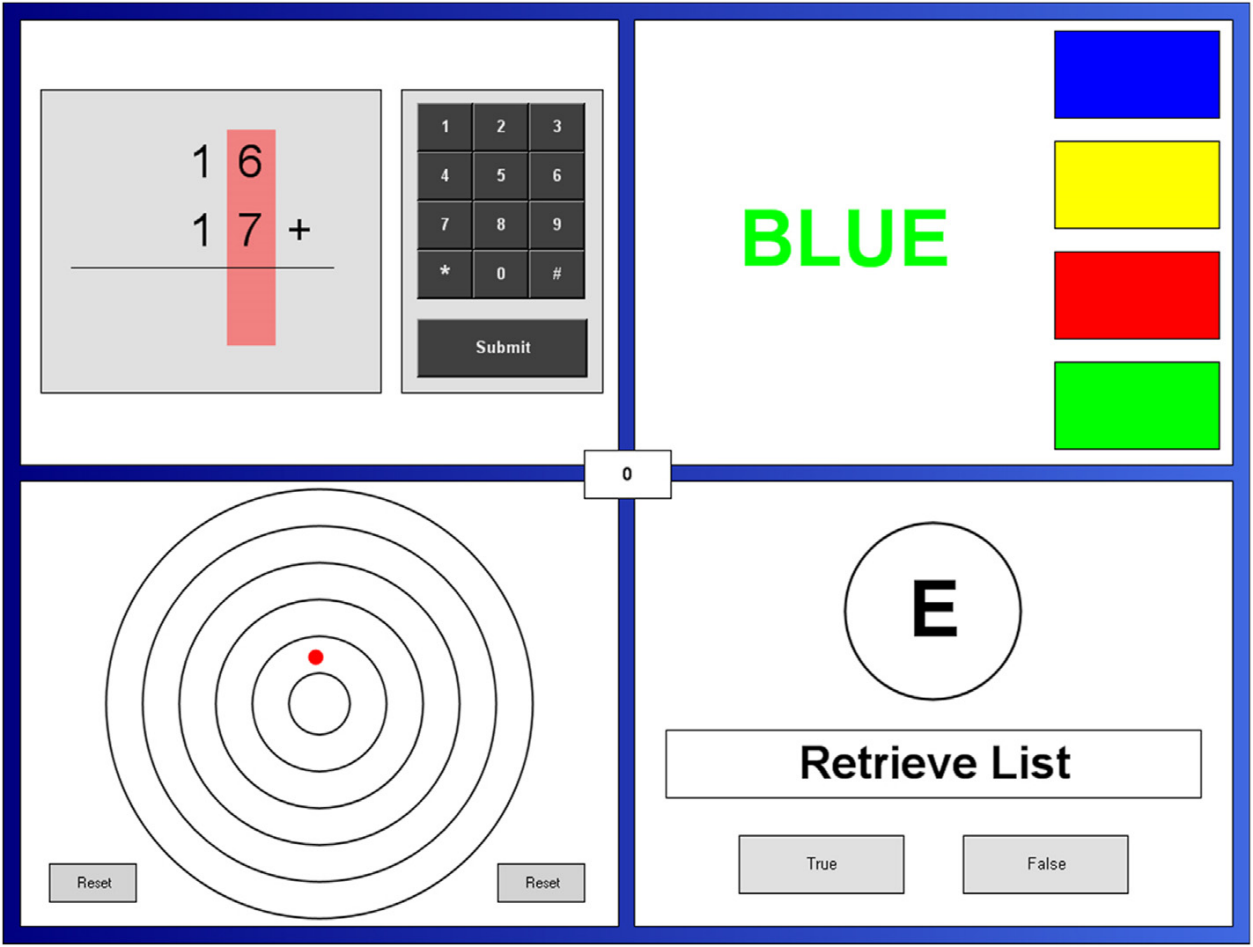
Screen layout of the Purple multitasking framework (MTF). The MTF requires participants to simultaneously perform 4 cognitive tasks that were (clockwise from top left) mental arithmetic, Stroop, letter recall, and visual tracking.

The MTF (Purple Research Solutions) was used for 2 purposes in this trial: acting as a cognitive stressor to allow for stress reactivity measurements and assessment of cognitive function under increased cognitive demand. The MTF comprises 4 tasks presented in quadrants of a split screen and requires concurrent attention to all tasks (Figure 2). In the center of the screen, a counter displaying the overall performance score based on accuracy and reaction time is presented. A 20-min version of the MTF was used, with mental arithmetic, Stroop, letter retrieval, and visual tracking tasks set to the medium intensity level. Participants were actively monitored by research staff at regular intervals throughout the test to heighten performance anxiety. The MTF has previously been shown to elicit cognitive demand, stress, negative mood, and anxiety [31] and is suitable for use in crossover trials with limited learning effects after several repetitions [32].

#### Cognitive performance

The MTF produces a concomitant assessment of memory, psychomotor, and attentional performance. A description of the 4 tasks and their respective scoring is summarized in Table 2. An aggregate total multitasking score was derived from the 4 individual tasks, reflective of executive functioning.

**TABLE 2:** Multitasking framework tasks and scoring.

MTF task	Description	Scoring
Mental arithmetic	Presents an addition equation of 2 rows of 3-digit numbers, requiring participants to enter their answer using the number pad	+10 points are allocated for correct responses and −10 points for incorrect responses, timeouts, or prompts
Stroop	Displays color words (blue, green, red, or yellow) in text that are presented in 1 of the 4 corresponding colors. Within 20 s, participants are required to indicate the font color by selecting the correct color displayed in the panel	
Letter recall	Displays a set of 4 letters that disappear after 4 s at the start of the test. A series of single probe letters are then presented. Participants are required to indicate whether the probe letter appeared in the original set of 4 letters	
Visual tracking	A red dot moves outward through a series of concentric circles. Participants are required to select the reset button before the red dot move beyond the outermost circle	A maximum of +10 points if the reset button is selected while the red dot is within the outermost circle, through to +2 points if within the innermost circle. Points are lost at a rate of −10 points for every 0.5 s where the red dot has reached the boundary of the outermost circle

#### Stress reactivity

Participants were asked to complete questionnaires [State Trait Anxiety Inventory—State Portion (STAI-S), Bond–Lader visual analog mood scale (VAMS), and stress and fatigue VAMS] asking them how they felt in the present moment immediately before and after completing the MTF. A change score (post-MTF − pre-MTF) was calculated to measure the subjective change in mood in response to the cognitive stressor. The questionnaire set has previously been used to measures stress reactivity in response to cognitive demand [26].

##### Spielberger STAI-S

The Spielberger STAI-S comprises 20 items (e.g., “I feel calm”) on a 4-point scale ranging from “not at all” to “very much so.” Scores were combined to give a range from 20 to 80, with higher scores indicating greater levels of present-state anxiety.

##### Bond–Lader VAMS

The Bond–Lader VAMS comprises 16 items, with a 100-mm line separating 2 antonyms anchored at either end (e.g., tense–relaxed). Participants indicated their current subjective state between the 2 antonyms on the line, with individual item scores calculated as the distance along the line from the negative antonym. The item scores were combined to form the 3 domains of alertness, calmness, and contentment, with higher scores indicating more positive mood states.

##### Stress and fatigue visual analog scales

Participants were asked to rate their current subjective experience of stress and fatigue on single 100-mm visual analog scales with the words extremely and not at all anchored at either end. Individual item scores were calculated as the distance along the line from the negative antonym, with higher scores indicating higher levels of stress or fatigue.

### Mood—POMS

The POMS questionnaire comprises 65 mood-related adjectives and required participants to indicate the degree to which they identified with each item over the past week on a 5-point scale from not at all to extremely. Six dimensions were derived from the 65 items, including tension/anxiety, confusion/bewilderment, anger/hostility, depression/dejection, fatigue/inertia, and vigor/activity. A total mood disturbance was also computed as the sum of the first 5 domains minus the vigor/activity score. Lower scores indicate better mood states, except in the case of vigor/activity where a high score is favorable [33].

### Sleep—PSQI

The PSQI questionnaire comprises 19 items that are grouped into 7 component scores including subjective sleep quality, sleep latency, sleep duration, habitual sleep efficiency, sleep disturbance, use of sleep medications, and daytime dysfunction. Each component was scored from 0 to 3, with a composite global PSQI generated by summing the component scores. The global PSQI score was reported in this study with a maximum score of 21, with higher scores indicating poorer sleep quality [34].

### Biochemistry analysis

Fasting venous blood samples were collected by a qualified phlebotomist (NAG) into 10-mL serum separator evacuated tubes and allowed to clot at room temperature for ≥15 min before being centrifuged at 2000 × *g* for 15 min at 4 °C. All samples were processed within 2 h of collection and then stored at −80 °C before delivery to a commercial laboratory for analysis (Liggins Institute). ELISA assays were used to measure serum kynurenine and tryptophan (ImmuSmol) and BDNF (Invitrogen) concentrations. A Cobas e601 autoanalyzer (Roche) was used to measure IL-6 concentrations by immunoassay. Coefficients of variance were below the acceptable 20% cutoff for each assay, based on ≥2 sets of quality controls analyzed alongside each plate. More than 95% of IL-6 samples were below the lowest detectable amount of 1.5 pg/mL, excluding IL-6 from any further reporting or statistical analysis.

### Gut microbiome profiling and bioinformatic analysis

Fecal samples were self-collected by participants 24 h before their study visits without preservative. Participants were instructed to store samples within their home freezer and transport them to their study visit with the provided ice packs. On receipt, samples were thawed and aliquoted, combining 100 mg stool in 900 μL of DNA/RNA Shield (Zymo Research International; #R1100) by vortexing. Samples were subsequently stored at −80 °C until DNA extraction was performed at the completion of the study. In total, DNA was extracted from 156 study samples, 3 ZymoBIOMICS Gut Microbiome Standards (Zymo Research International; # D6331), and 3 extraction blanks using the standard protocol for ZymoBIOMICS 96 MagBead DNA Kit (Zymo Research International; #D4308). Mechanical bead beating was performed on a 1600 MiniG (SPEX SamplePrep), shaking at full speed (1500 × *g*) for 5 min. Extract purity was assessed by spectrophotometry, using the Qubit dsDNA Broad Range Assay Kit (Invitrogen; #Q33266) to measure DNA concentration.

Metagenomic sequencing data were processed by KneadData (version 0.10.0) to trim and remove poor quality reads and those that mapped to the human genome (hg19). Taxonomic profiling was subsequently performed with MetaPhlAn3 (version 3.1). Functional profiling was performed with HUMAnN3 (version 3.6), with resulting gene families and pathway abundance tables renormalized to copies per million.

Downstream analysis and plot generation were conducted in R statistical software (version 4.2.1). The vegan package was used to calculate α-diversity (Shannon index) and β-diversity (Bray–Curtis dissimilarity) based on species-level composition. Ordinations were performed by nonmetric multidimensional scaling, and differences in microbiome structure between intervention arms were assessed by PERMANOVA. The MaAsLin2 package was used to test for differentially abundant species, genera and pathways using both general linear models and linear mixed-effects modeling designs. All models included a log transformation, minimum prevalence was set to 0.1, and the default significance threshold following false discovery rate correction was used (*q* < 0.25). To identify microbiome features that changed after consumption of the active beverage, models were fitted with fixed effects for intervention group (0 = placebo and 1 = active), time (0 = baseline and 1 = 4 wk), and their interaction (1 = active and 4 wk, 0 = all other combinations), and subject ID was added as a random effect.

### Statistical analysis

A power calculation was performed, based on previous reported mean changes in stress reactivity measures between an intervention containing similar doses of anthocyanins and a control [35]. Thirty-six participants would allow the detection of significant effects with a power of 0.8 at α = 0.05, with 40 participants recruited to allow for dropout.

Intention-to-treat analyses were conducted including all participants who received ≥1 treatment dose and satisfied the inclusion criteria at enrollment into the study. Statistical analyses were performed using R 4.2.1 Statistical Software [36], with *P* value of <0.05 considered significant unless otherwise specified. Before analysis, multiple imputations of missing data were conducted using chained equations in the MICE package, and distribution of continuous variables was graphically assessed for normality and outliers to ensure their appropriateness for parametric statistical tests.

Linear mixed-effect models were used to examine changes in stress reactivity, cognitive, mood, sleep, and blood measures from baseline to follow-up within and between intervention groups. The models included time (baseline and 4 wk), intervention group (active and placebo), their interaction (time × intervention group), and sequence allocation as fixed factors and subject ID as a random factor to account for repeated measures. Significant interaction effects were followed by multiple pairwise comparisons with Tukey adjustment.

Associations between gut microbiome features and clinical parameters were restricted to those clinical parameters shown to improve in the aforementioned analyses. This included analyses to identify differences in baseline gut microbiome features of clinical responders, using MaAsLin2’s linear mixed-effect models fitted with a binary response category (0 = nonresponder and 1 = responder). Responders were defined as those participants with changes to clinical scores in the top quartile.

## Results

### Participants

**FIGURE 3:**
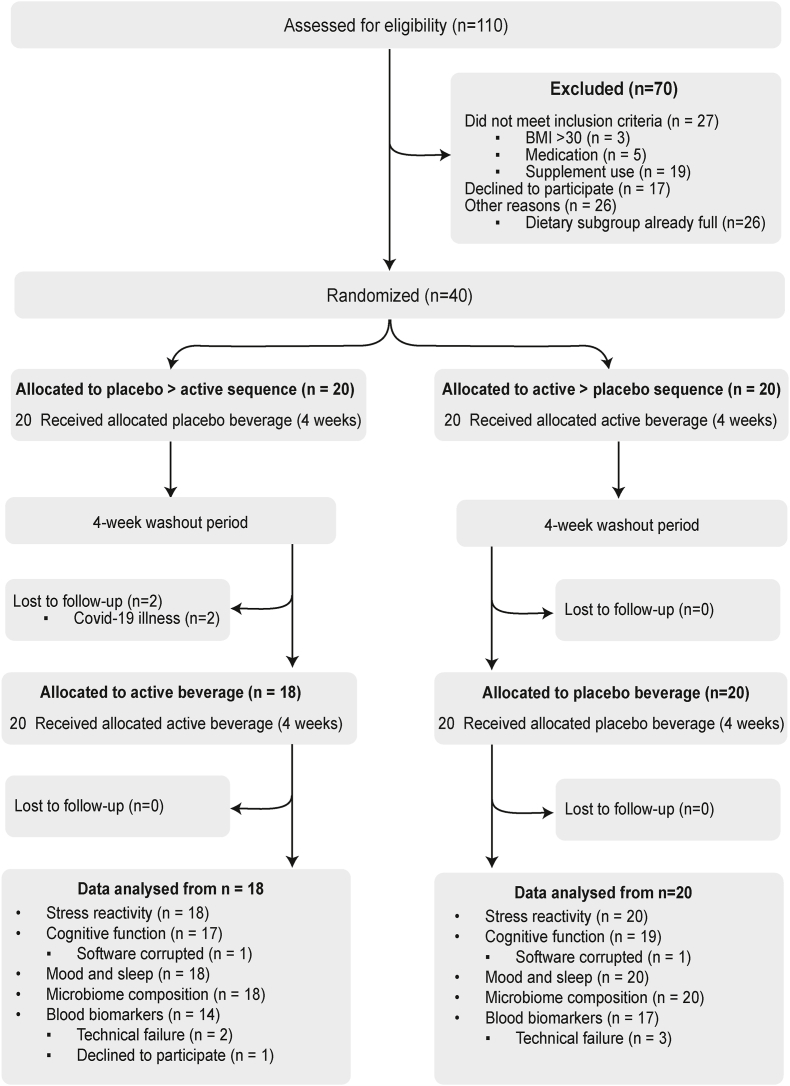
CONSORT flow diagram of the LINK study.

Of 110 potential participants screened, 40 healthy females were enrolled into the trial. A total of 38 participants (95% retention) completed both intervention arms in this crossover trial between July 2022 and January 2023, with 2 participants withdrawing before starting their second intervention arm for COVID-19–related reasons (Figure 3). As summarized in Table 3, this was a healthy population of females with a mean age of 29.8 y and most participants rating their own health as either excellent (37%) or very good (40%). Participants regularly engaged in moderate-intensity or high-intensity exercise, and the majority of participants were employed full time (63%) and had completed either undergraduate (47%) or postgraduate (45%) university degrees. Nutrient intake according to 3-d food records is presented in Supplemental Table 1, with group averages within recommended ranges for most nutrients with the exception of calcium and sodium.

**TABLE 3:** Characteristics of participants who completed both intervention arms in the LINK trial.

	Total sample (*n* = 38)	Active > placebo sequence (*n* = 20)	Placebo > active sequence (*n* = 18)
Age (y)	29.8 ± 7.1	30.2 ± 8.0	29.5 ± 6.0
BMI (kg/m^2^)	22.8 ± 3.3	23.3 ± 4.1	22.1 ± 1.9
Alcohol (drinks/wk)	2.6 ± 2.8	3.2 ± 3.0	1.9 ± 2.4
Supplement use			
Yes	8 (21.1)	5 (25)	6 (33)
No	30 (78.9)	15 (75)	12 (67)
Education			
Secondary school	3 (7.9)	2 (10)	1 (6)
University: undergraduate	18 (47.4)	10 (50)	8 (44)
University: postgraduate	17 (44.7)	8 (40)	9 (50)
Exercise^1^			
High	21 (56.8)	9 (45)	12 (67)
Moderate	14 (36.8)	10 (50)	4 (22)
Low	3 (8.1)	1 (5)	2 (11)
Self-rated health			
Excellent	14 (36.8)	7 (35)	7 (39)
Very good	15 (39.5)	7 (35)	8 (44)
Good	8 (21.1)	5 (25)	3 (17)
Fair/poor	1 (2.6)	1 (5)	0 (0)
Dietary screening tool (DST)			
Total score	65.8 ± 12.6	65.7 ± 12.7	65.9 ± 12.9
Optimal group (DST > 60)	19 (50)	9 (50)	10 (50)
Suboptimal group (DST ≤ 60)	19 (50)	9 (50)	10 (50)

Values are mean ± SD or *n* (%).

1 Exercise intensity was estimated from the International Physical Activity Questionnaire—short form.

All participants met minimum compliance requirements of 80% (Supplemental Figure 1). A summary of adverse events reported during the intervention period is provided in Supplemental Table 2. In summary, the frequency of reported adverse events was similar between active (18%) and placebo (27%) beverages, with the majority of adverse events being categorized as mild (82% of reported adverse events), and gastrointestinal symptoms were the most commonly reported adverse event (82% of all reported adverse events).

### Primary outcome—stress reactivity

There was no difference in changes of stress reactivity measures (alertness, calmness, contentedness, stress, and fatigue) between intervention groups after 4 wk of intervention with active and placebo beverages, including scores from the Bond–Lader VAMS, fatigue and stress visual analog scales, and the STAI-S tools. Raw data and effect size estimates derived from linear mixed-effect models are presented in Table 4.

**TABLE 4:** Change in clinical parameters rom baseline to follow-up according to intervention group.

Outcome	Measure/domain	Active intervention			Placebo control			Difference in change betweenactive and placebo	
		Baseline	4 wk	Δ	Baseline	4 wk	Δ	Effect size	*P*
Stress reactivity	Bond–Lader VAMS								
	Alertness	0.3 ± 1.1	0.5 ± 1.0	0.2	0.1 ± 1.4	0.2 ± 1.3	0.1	0.1	0.723
	Calmness	−1.5 ± 2.3	−1.1 ± 1.8	0.4	−1.6 ± 1.7	−1.3 ± 1.9	0.3	0.1	0.893
	Contentedness	0.3 ± 0.9	0.0 ± 0.8	−0.2	0.3 ± 0.8	0.2 ± 1.0	−0.1	0.1	0.821
	Fatigue VAS	0.4 ± 2.0	0.2 ± 1.5	−0.2	0.6 ± 1.6	0.3 ± 1.6	−0.3	0.0	0.999
	Stress VAS	0.3 ± 1.9	0.2 ± 1.8	−0.1	0.8 ± 2.1	0.5 ± 1.9	−0.3	0.2	0.749
	STAI-S	1.6 ± 6.7	1.7 ± 5.2	0.1	2.6 ± 5.1	1.8 ± 4.9	0.8	0.9	0.539
Cognition	Mental arithmetic	440 ± 311	462 ±331	22	423 ±353	505 ± 341	82	60	0.383
	Letter retrieval	3098 ± 1924	4124 ± 2036	1026	3485 ± 1665	3853 ± 1985	368	658	0.034^1^
	Stroop	2750 ± 1995	3158 ± 2178	408	2933 ± 1632	3221 ± 1463	288	121	0.773
	Visual tracking	406 ± 255	448 ± 56	42	441 ± 88	429 ± 131	12	53	0.551
	Total MTF score	6696 ± 2782	8205 ± 2702	1509	7281 ± 2455	8004 ± 2106	723	785	0.182
Mood	Anger	5.4 ± 4.8	3.4 ±3.2	−2.0	4.2 ±3.5	4.0 ± 4.2	−0.2	1.8	0.043^2^
	Confusion	7.0 ± 2.9	5.8 ± 2.1	−1.2	6.7 ±2.7	6.4 ±2.7	−0.3	0.9	0.126
	Depression	5.8 ±6.1	3.8 ±4.4	−2.0	6.5 ± 6.0	6.0 ± 7.2	−0.5	1.5	0.293
	Fatigue	9.0 ± 4.4	7.1 ± 4.9	−1.9	8.9 ± 5.5	8.7 ±5.1	−0.2	1.8	0.100
	Tension	8.6 ± 4.0	6.9 ± 3.6	−1.7	8.0 ± 4.0	8.1 ±4.5	0.1	1.9	0.030^3^
	Vigor	15.1 ±5.5	15.3 ± 5.5	0.2	14.1 ±5.7	14.1 ± 6.7	0.0	0.1	0.920
	Total mood disturbance	20.6 ± 20.5	11.7 ± 16.3	−8.9	20.1 ± 20.7	19.1 ±23.7	−1.0	8.0	0.052^4^
Sleep	PSQI	5.8 ±.9 2.9	5.0 ± 2.9	−0.8	6.0 ±2	5.6 ± 3.0	−0.4	0.4	0.441
Biochemical marker	BDNF (pg/mL)	25,676 ± 6885	25,959 ± 6661	283	27,068 ± 8820	26,716 ± 7235	−352	636	0.727
	Tryptophan (μg/mL)	11.3 ± 2.8	11.0 ± 2.6	−0.3	11.7 ± 2.4	11.2 ± 2.6	−0.5	0.2	0.715
	Kynurenine (ng/mL)	457 ± 86	480 ± 160	23	469 ± 98	457 ± 95	−12	36	0.265
	Tryptophan/kynurenine	0.03 ± 0.01	0.02 ± 0.01	0.0	0.03 ± 0.01	0.03 ± 0.01	0.0	0.0	0.683

Abbreviations: BDNF, brain-derived neurotrophic factor; PSQI, Pittsburgh Sleep Quality Index; STAI-S, State trait anxiety inventory—stress subscale; VAMS, visual analog mood scale; VAS, visual analog scale.

Effect sizes (estimated marginal means of differences between groups) and *P* values are derived from linear mixed-effect models. According to post hoc analyses with Tukey adjustment for multiple comparisons on variables with significant interaction effects, the following results were found:

1 Letter retrieval scores increased with the active intervention (*P* < 0.001) but not placebo (*P* = 0.331).

2 Perceived anger levels improved with the active intervention (*P* = 0.013) but not placebo (*P* = 0.997).

3 Perceived tension levels improved with the active intervention (*P* = 0.023) but not placebo (*P* = 0.998).

4 Given the closeness to significance threshold, post hoc analyses were performed and revealed that total mood disturbance improved with the active intervention (*P* = 0.013) but not placebo (*P* = 0.989).

### Secondary outcomes

#### Cognitive performance

**FIGURE 4:**
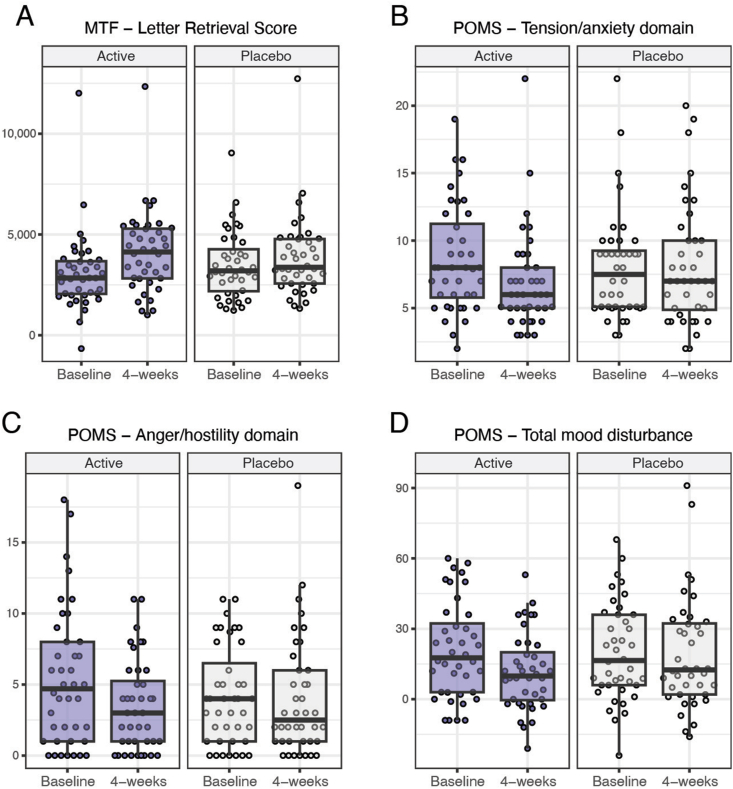
Change in cognitive and mood measures differ between intervention groups. Post hoc analyses of significant time × intervention group interactions revealed marked improvements following 4 wk of the active intervention for the following: (A) letter retrieval scores: Δ = 1026-point increase; *P* < 0.001, but not placebo (*P* >.05); (B) tension/anxiety: Δ = 1.7-point reduction, *P* = 0.023; (C) anger/hostility: Δ = 19-point reduction; *P* = 0.013; (D) total mood disturbance: Δ = 8.9-point reduction; *P* = 0.013; however, note a near-significant interaction effect for this domain (*P* = 0.052). MTF, multitasking framework; POMS, profile of mood states.

Changes (Δ = 4-wk score – baseline score) in letter retrieval domain scores from the MTF, a measure of working memory, were significantly different between intervention groups (*P*-interaction = 0.034) (Table 4). Multiple pairwise comparisons with Tukey adjustment showed a 33% improvement in letter retrieval scores after 4 wk of the FBB (Δ = 1026-point increase; *P* < 0.001), whereas no change was observed with the placebo beverage (Δ = 368-point increase; *P* = 0.331) (Figure 4).

#### Mood

Changes in anger/hostility and tension/anxiety domains of the POMS questionnaire differed between intervention groups (anger: *P*-interaction = 0.043; tension: *P*-interaction = 0.030) (Table 4). Multiple pairwise comparisons with Tukey adjustment showed 37% less anger (Δ = 1.9-point reduction; *P* = 0.013) and 20% less tension (Δ = 1.7-point reduction; *P* = 0.023) in POMS scores after 4 wk of the FBB, whereas no change was observed with the placebo beverage (*P* > 0.05) (Figure 4). The interaction effect neared significance for total mood disturbance scores (*P* = 0.052), with multiple pairwise comparisons showing a 43% improvement after 4 wk of the active intervention (Δ = 8.9-point reduction; *P* = 0.013) but not in the placebo (Δ = 0.9-point reduction; *P* = 0.989).

#### Sleep and biochemical markers

There were no differences in changes to global PSQI scores or any of the biochemical markers under investigation (BDNF, tryptophan, kynurenine, and tryptophan/kynurenine ratio) between intervention groups (Table 4).

#### Microbiome composition

**FIGURE 5:**
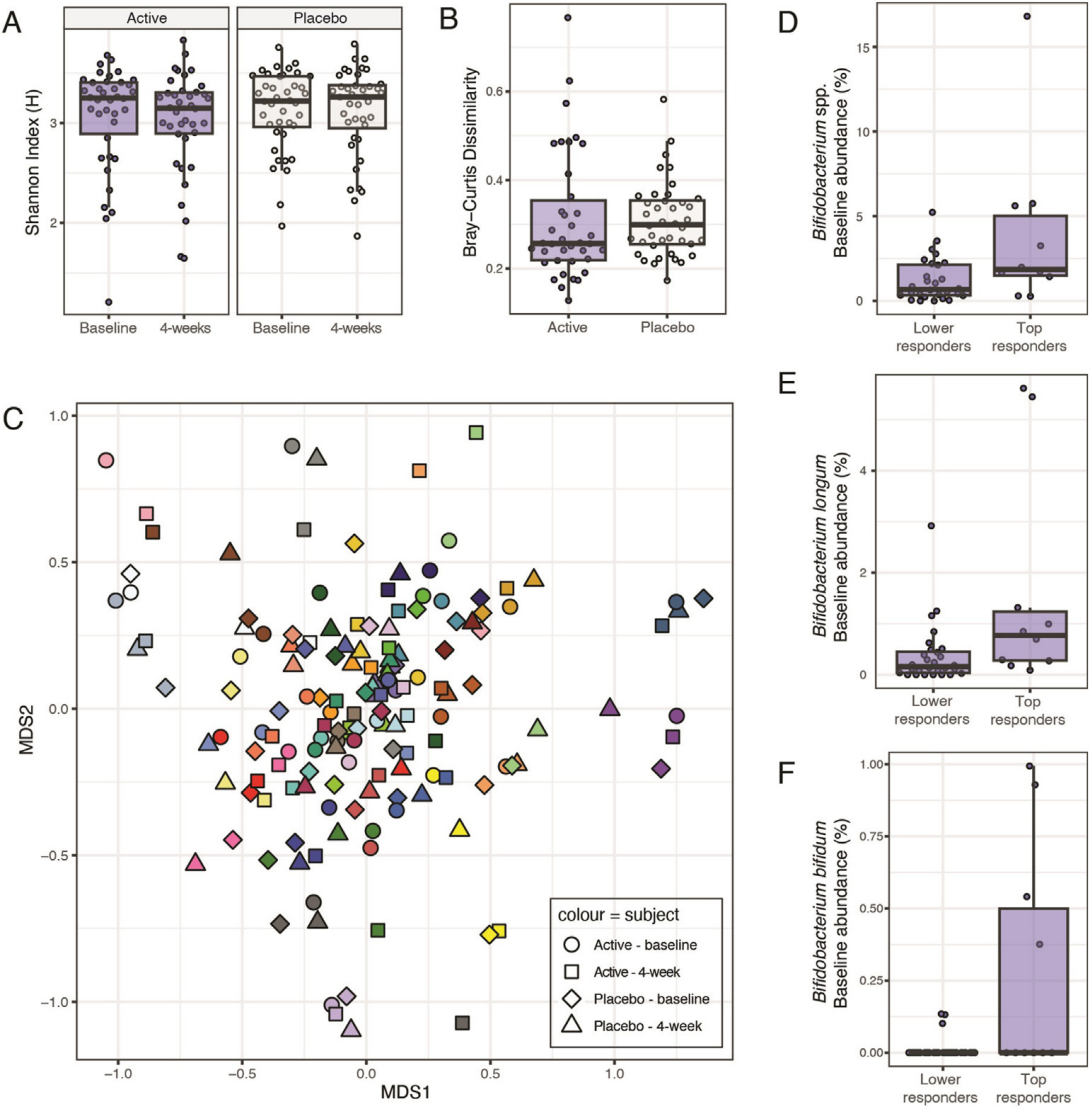
Effect of the PBB intervention on gut microbiome composition. (A) Shannon diversity index based on species composition at baseline and 4 wk after supplementation with active and placebo beverages. (B) Bray–Curtis dissimilarity in species composition between participant’s baseline and 4-wk samples for both intervention arms. (C) Nonmetric multidimensional scaling (MDS) plot showing that variability in species composition was largely driven by subject rather than by intervention. Relative abundance of the genus *Bifidobacterium* genera (D) and *Bifidobacterium longum* (E) and *Bifidobacterium bifidum* (F) was higher at baseline in the top quartile of participants whose letter retrieval scores improved (i.e., the top responders).

There were no differences in α-diversity metrics (Shannon index), nor in the number of detected species, gene families, or pathways between intervention arms (Figure 5A). There was also no difference in microbiome composition shifts between the intervention arms, comparing participant baseline and 4-wk samples (Wilcoxon rank sum test, *P* = 0.21) (Figure 5B). Similarly, there were no intervention effects on β diversity (PERMANOVA, *R*^2^ = 0.45%; *P* = 0.81), with microbiome samples instead clustering by participant (PERMANOVA, *R*^2^ = 79.5%; *P* = 0.001) (Figure 5C).

The next analysis tested whether there were any specific species, genera, or microbiome encoded pathways whose relative abundance changed after supplementation with the FBB. Thirteen species, 3 genera, and 7 pathways were differentially abundant based on a significance threshold of *P* < 0.05; however, none of these remained significant after adjusting for multiple testing (*q* > 0.25) (Supplemental File 2).

#### Microbiome features associated with clinical response

We explored whether there were any baseline gut microbiome features that differentiated participants who had greater improvements (responders defined as the top quartile) in behavioral outcomes. This analysis was restricted to clinical variables shown to significantly improve with FBB supplementation in previous analyses, including letter retrieval, tension/anxiety, and anger/hostility scores.

Although there were no differences in α-diversity or richness (at species and gene levels), there was a nominally significant higher abundance of *Bifidobacterium* spp, at baseline who exhibited greater improvements in letter retrieval scores (Figure 5D), specifically *Bifidobacterium longum* and *Bifidobacterium bifidum* (MaAsLin2 model; *P* < 0.05) (Figure 5E, F). A full summary of MaAsLin2 models identifying differences in species, genera, and pathways between responders and nonresponders for letter retrieval, tension/anxiety, and anger/hostility outcomes is provided in Supplemental File 2. Partial bivariate Pearson correlation analyses were planned to assess associations between changes in microbiome features and clinical parameters, but this was not carried out given that there were no significant change in species, genera, or pathways as a result of the FBB.

## Discussion

The LINK study investigated the effects of an FBB on markers of the microbiota–gut–brain axis. This randomized, double-blind, placebo-controlled, crossover trial found that, compared with a placebo drink, daily consumption of the FBB for 4 wk improved secondary outcomes of cognitive performance during multitasking and mood in a group of healthy female adults. Although there were no changes to gut microbiome composition or functional potential as a result of the FBB, there was a nominal difference in baseline relative abundance of *Bifidobacterium* genera and species (*B. longum* and *B. bifidum)* participants who had greater improvements in cognitive responses following the FBB supplementation.

Stress reactivity refers to the way in which people respond to stressful or demanding situations, specifically in this case where cognitive resources are insufficient to meet ongoing task demands. It is a mechanism by which psychological stress is proposed to contribute to health or disease, including mental health and neurologic disorders [22,23]. Kimble et al. [35] have shown that 3 mo supplementation with anthocyanin-rich tart cherries improves measures of stress reactivity in middle-aged adults, including fatigue and mental alertness, following a cognitive battery. Although no such improvements were found in our primary outcome of stress reactivity with the FBB, it is important to note that our participants were minimally responsive to the MTF as a cognitive stressor. This is despite using the same MTF protocols that have reliably increased self-ratings of negative mood and anxiety as well as psychobiological stress responses [31]. Although the presence of learning effects is a possibility, previous research has shown that the MTF can be repeated on multiple occasions without habituation of responses [32]. Moreover, given only trivial changes in scores seen in this study, a learning effect is unlikely. Findings from this study therefore cannot confirm or refute whether the FBB buffers psychological stress in response to situations perceived as stressful or demanding, as any potential intervention effect would have been masked by the lack of response to the MTF. Interestingly, acute blackcurrant supplementation has been shown to alter electroencephalogram spectral power activity in healthy young adults during a 21-min cognitive battery despite no change to subjective mood questionnaires, including evidence of an anxiolytic effect from increased slow wave Δ and θ spectral powers [37]. Using more objective measures of stress (e.g., salivary cortisol or heart rate monitoring) would have been helpful to confirm participants’ lack of subjective response reported in this study.

The improvements to cognitive performance observed after 4 wk of daily FBB intake are consistent with previous evidence describing cognitive benefits of supplementation with anthocyanin-rich fruits [11,35,38,39] and flavonoid-rich foods or beverages [40,41]. Memory appears to be a specific cognitive domain sensitive to flavonoid supplementation [42], although direct comparisons to this literature are somewhat limited by differences in cognitive testing paradigms. Our results do require interpretation in the context of how cognitive function was evaluated. Multitasking while being monitored is an ecologic behavior with clear parallels in day-to-day cognition. It requires integration of several complex processes across different cognitive domains [31]. Although improvements to the letter recall task do relate to working memory function, this cannot be isolated from attention and executive functioning, both being required to perform throughout the multitasking paradigm. Regardless, it is promising to see benefits for performance in healthy, younger populations, which is a critical time for brain health optimization and prevention of disease [43].

There was also improvements in tension/anxiety, anger/hostility, and total mood disturbance scores of the POMS questionnaire after 4 wk of FBB supplementation, with a magnitude of 20%–43% improvement across the domains. Although polyphenols target multiple pathophysiologic pathways relevant to both mental health and cognition [5,12], the evidence base in human intervention trials is much more limited in the relatively nascent field of nutritional psychiatry than that for cognitive function. Previous flavonoid interventions have typically been conducted in disease states like depression [12] or in acute intervention settings [39,44], although Fisk et al. [45] have shown improvements to mood with 4 wk of wild blueberry supplementation (253 mg anthocyanins) in healthy adolescents aged 12–17 y. To the best of our knowledge, this is the first time that improvements to mood have been observed in a healthy adult population for any of the constituent ingredients or bioactives. Although there appeared to be consistency in changes across several domains of mood observed in this study, cautious interpretation is needed given that these were secondary outcomes unadjusted for multiple testing. These findings warrant future investigation in a study adequately powered for such outcomes and with a longer-term follow-up to understand whether the effects are true or spurious.

We investigated blood biomarkers relevant to the gut–microbiota–brain axis in attempt to gain mechanistic insights but found no changes to BDNF or the kynurenine/tryptophan ratio, and the majority of our healthy participants had inflammatory markers below the detectable limit, which prevented further analysis. BDNF is a critical modulator of synaptic plasticity, with previous literature showing increased BDNF concentrations following polyphenol supplementation in human intervention trials [46,47] and preclinical research [48]. Although no change was reported in this study, the testing conditions might have contributed to this. For example, stress reactivity and cognitive measures were prioritized and performed before the blood draw, which might have itself resulted in changes in biomarkers. Additionally, the tryptophan/kynurenine ratio may be more sensitive to changes in disease conditions, as previous research has shown an attenuated increase in the kynurenine/tryptophan ratio in a mouse model of agitated depression with flavonoid supplementation [49], although there are limited human intervention trials investigating the markers of the kynurenine pathway in response to flavonoid supplementation [12].

Modulating the gut microbiome is hypothesized as a mechanism by which mood [5] and cognitive [50] benefits of polyphenols are mediated. Dietary polyphenols are proposed to have a prebiotic-like effect, stimulating the abundance of health-promoting species such as *Bifidobacteria* spp and *Lactobacillus* spp and inhibit the growth of potentially pathogenic taxa such as *Clostridium* spp [51]. Our data did not support a prebiotic effect of the FBB, although this is perhaps unsurprising given that this was a healthy population with generally high-fiber intakes (29 ± 13 g/d). Furthermore, the gut microbiome is highly variable between individuals, which makes it difficult to find consistent changes associated with nutritional interventions, particularly with a small sample size [24,52]. We might expect to see a more pronounced response in conditions characterized by dysbiosis. For example, supplementation with red wine polyphenols for 30 d has been shown to increase relative abundance of *Bifidobacteria* and *Lactobacillus* spp in patients with metabolic syndrome but not healthy controls [53].

Although there was no significant changes to the relative abundance of microbial taxa or diversity metrics in response to the FBB, this does not exclude the possibility that the cognitive and mood benefits observed in this study are still mediated through the gut microbiome. For example, in vitro work has shown the ability of anthocyanins to increase short-chain fatty acid production [54,55], organic acids that are known to influence cognition and mood [56]. It may be that the underlying structure and encoded functions of the microbiome remain stable, as seen in this study with microbial gene abundance data, but its activity is altered. Future research should consider analyses of the actively transcribed genes (meta-transcriptomics) or gut-derived metabolites and metabolome (standard analytical chemistry methods and metabolomics) to better understand the role of the gut microbiome in mediating the clinical benefits of polyphenol supplementation.

Although research on cardiometabolic or cognitive effects of polyphenol supplementation are now beginning to include gut microbiome measurements alongside clinical parameters [18,19], few have considered baseline gut microbiome as a mechanistic key to understanding clinical effects. To the best of our knowledge, this is the first human intervention trial that has shown differences in baseline gut microbiome composition in cognitive responders. Specifically, we found that our clinical responder group (in the top quartile of response) for letter recall scores had nominally higher concentrations of *Bifidobacterium* spp, at baseline, including *B. longum* and *B. bifidum.* We propose that these findings implicate a role for *Bifidobacterium* spp in moderating how participants responded to the FBB. This aligns with previous in vitro [54] and human clinical research [57], showing that bacteria from the *Bifidobacterium* genus have the capacity to metabolize anthocyanin extracts into small molecular phenolic acids. Indeed, microbial biotransformation has been proposed as a key step in the ability of polyphenols to exert clinical benefits [15,16]. The relevance of our findings for future research lies in more targeted (e.g., gut microbiome profiling) intervention strategies to reveal the true potential of polyphenols in a field with highly variable findings or enhanced intervention strategies (e.g., cosupplementation with relevant probiotic species). Larger-scale intervention trials with more targeted and diverse polyphenol treatments are required to prove this concept.

This study was a rigorous, double-blinded, crossover trial with excellent participant retention and adherence; however, this study should be evaluated in the context of its limitations. First, the commercially available FBB investigated in this study contained a blend of bioactive ingredients. This includes 151 mg anthocyanins from blackcurrant juice and extracts, 150 mg proanthocyanidin-rich *P. radiata* extract contributing toward the total polyphenol dose of 308 mg, and 200 mg L-theanine. The observed effects can therefore not be attributed to any single ingredient. This is also an issue for polyphenol-rich foods or beverages where other bioactive compounds are present, a good example being caffeine in cocoa or vitamin C content in fruits. We have discussed that our findings align with previous research on anthocyanin-rich fruits and the literature on flavonoids more broadly, but the fact that L-theanine is not an inert ingredient is an important limitation. The neuropharmacologic effects of L-theanine are well-defined, including an influence on brain wave oscillations and neurotransmitter synthesis and degradation [58], with a net effect of reduced excitatory activity and enhanced performance under conditions of attention or stress [59]. Translation of efficacy to human clinical intervention trials have been much less clear however, and the majority of research has been performed in acute settings [60,61] compared with evidence from flavonoid intervention studies where more convincing effects are seen with longer-term studies [10]. Given that L-theanine peaks in plasma 50 min after ingestion and is largely cleared from plasma within 24 h, it is unlikely that L-theanine is driving the results observed in this study. Analyzing urinary or fecal polyphenol metabolites and correlating with clinical outcomes could have helped to overcome this issue. Having a third arm in the study (e.g., FBB with and without L-theanine) would also be an interesting experiment to understand whether bioactives, such as L-theanine can enhance or interact with polyphenols [60] but was beyond the scope of this research, which primarily focused on understanding interactions with the microbiota–gut–brain axis where the influence of L-theanine was considered negligible.

Another important limitation is that our dietary assessment methodology does not capture habitual polyphenol intake, although this is widely acknowledged as a challenge in the research field owing to inadequate dietary assessment tools and limited locality-specific food content data [12,62]. Some studies do attempt to isolate intervention effects by restricting background polyphenol intake [35]. We also did not conduct follow-up dietary assessment, and although participants reported that they had not made any changes to diet/lifestyle, which was crosschecked with researchers, we cannot be absolutely certain that dietary changes may have contributed to intervention effects. Finally, females were selected as the study population given their greater risk of mental health disorders [63] and conditions relevant to microbiota–gut–brain axis dysfunction like irritable bowel syndrome [64,65] but this does of course limit generalizability of findings. The clinical relevance of our findings is also limited by the younger age range (18–45 y) and health of our participants and relatively short 4-wk intervention period. We also acknowledge inflated risk of type 1 error because of the number of outcomes measured and statistical tests conducted, but these were predefined and conducted with appropriate correction in the case of multiple pairwise comparisons. It is pertinent to highlight that we have not adjusted to a more conservative level of significance for the number of secondary outcomes tested. Although this approach does generate pathways for future research such as in the case of mood effects observed in this study, this does necessitate cautious interpretation of this study.

In conclusion, this study is a novel investigation into the effects of a FBB on markers of the microbiota–gut–brain axis in healthy female adults. We found that daily consumption of the FBB for 4 wk improved secondary outcomes of cognitive function and mood. Although the FBB did not alter gut microbiome composition or diversity, participants with the greatest cognitive responses had a greater relative abundance of *Bifidobacterium* spp, at baseline which implicates the gut microbiome in mediating the benefits of the flavonoid-rich intervention. This research adds to a growing body of evidence, which supports flavonoids as a plausible preventative strategy to tackle the burden of interrelated brain and mental health disorders, but improvements to secondary outcomes observed should be confirmed in larger sample sizes with a longer intervention period. Future research is needed to understand whether enhanced intervention effects can be observed with a more targeted approach through gut microbiome profiling at baseline or perhaps with concurrent probiotic supplementation.

## Author contributions

The authors’ responsibilities were as follows – NAG, NCR, AS, AJB: designed the research; NAG, BCW: conducted research; NAG, BCW, JRM: analyzed data; NAG, BCW: wrote the paper; NAG: had primary responsibility for final content; and all authors read and approved the final manuscript.

## Conflict of Interest

In the last 3 y, AS has received research funding and/or consultancy/travel/speaker fees from BioRevive, Coca Cola, Ekterra, Frutarom, Givaudan, National Health and Medical Research Council, Nestlé, Nutricia Research Foundation, PepsiCo, Reviv, Wörwag Pharma, and Wrigley-Mars. AS is Chief Scientific officer for Ārepa. All other authors report no conflicts of interest.

## Funding

This research was funded by the New Zealand National Science Challenge (High-Value Nutrition, #1968). The funders had no role in the study design, collection, analysis or interpretation of data, or writing the manuscript. There were no restrictions regarding submission of the manuscript for publication. All intervention and placebo beverages were contributed in-kind by Ārepa IP.

## Data availability

Deidentified data described in the manuscript, code book, and analytic code will be made available on reasonable request via e-mail to the corresponding author.
