# Supplementary material for: Effects of a Flavonoid-Rich Blackcurrant Beverage on Markers of the Gut-Brain Axis in Healthy Females: Secondary Findings From a 4-Week Randomized Crossover Control Trial
Source: Curr Dev Nutr. 2024 Apr 12;8(5):102158. doi: 10.1016/j.cdnut.2024.102158 (PMC11074983; doi:10.1016/j.cdnut.2024.102158)
Supplement: Multimedia component 1 [file mmc1.docx]

**Supplementary Table 1. Habitual dietary intake analyzed from 3-day food records**

|  | **EAR or AI^1^** | **Total sample (n=38)** | **Optimal (n=19)** | **Sub-optimal (n=19)** |
| --- | --- | --- | --- | --- |
|  |  |  |  |  |
| Energy (kJ) | - | 7985 ± 1897 | 7927 ± 1786 | 8042 ± 2050 |
| Protein (g) | - | 81 ± 20 | 78 ± 18 | 84 ± 21 |
| kJ from protein (%) | - | 18 ± 4 | 17 ± 2 | 19 ± 5 |
| Total fat (g) | - | 87 ± 29 | 84 ± 26 | 90 ± 31 |
| kJ from fat (%) | - | 40 ± 8 | 39 ± 6 | 42 ± 9 |
| Saturated fat (g) | - | 32 ± 15 | 29 ± 9 | 34 ± 20 |
| kJ from saturated fat (%) | - | 15 ± 5 | 14 ± 3 | 16 ± 7 |
| Polyunsaturated fat (g) | - | 15 ± 6 | 14 ± 6 | 15 ± 6 |
| Monounsaturated fat (g) | - | 32 ± 11 | 32 ± 13 | 31 ± 9 |
| Total carbohydrate (g) | - | 183 ± 64 | 189 ± 51 | 177 ± 76 |
| kJ from carbohydrate (%) | - | 38 ± 10 | 40 ± 6 | 37 ± 13 |
| Sugar (g) | - | 74 ± 27 | 76 ± 20 | 72 ± 34 |
| Fibre (g) | 25 | 29 ± 13 | 31 ± 11 | 27 ± 15 |
|  |  |  |  |  |
| Calcium (mg) | 840 | 799 ± 297 | 791 ± 287 | 806 ± 314 |
| Iodine (μg) | 100 | 121 ± 127 | 100 ± 48 | 142 ± 174 |
| Iron (mg) | 8.0 | 12.2 ± 4.4 | 12.9 ± 5.2 | 11.6 ± 3.4 |
| Magnesium (mg)^2^ | 255-265 | 357 ± 130 | 373 ± 122 | 341 ± 139 |
| Phosphorus (mg) | 580 | 1333 ± 328 | 1333 ± 303 | 1334 ± 361 |
| Potassium (mg) | 2800 | 3192 ± 1157 | 3271 ± 1067 | 3112 ± 1264 |
| Selenium (μg) | 50 | 56 ± 26 | 56 ± 26 | 57 ± 25 |
| Sodium (mg) | 460-920 | 2331 ± 902 | 2151 ± 769 | 2512 ± 1006 |
| Zinc (mg) | 6.5 | 9.8 ± 2.7 | 9.2 ± 2.4 | 10.4 ± 2.9 |
|  |  |  |  |  |
| Vitamin A (μg) | 500 | 1063 ± 1048 | 958 ± 418 | 1168 ± 1436 |
| Beta carotene (μg) | - | 3850 ± 2681 | 4224 ± 2447 | 3476 ± 2914 |
| Thiamin (mg) | 0.9 | 1.2 ± 0.7 | 1.1 ± 0.5 | 1.3 ± 0.8 |
| Riboflavin (mg) | 0.9 | 1.6 ± 0.7 | 1.5 ± 0.4 | 1.7 ± 0.8 |
| Niacin (mg) | 11 | 17.3 ± 6.6 | 16.9 ± 6.1 | 17.6 ± 7.2 |
| Vitamin B_6_ (mg) | 1.1 | 2.2 ± 1.1 | 2.2 ± 0.7 | 2.3 ± 1.5 |
| Folate DFE (μg) | 320 | 420 ± 180 | 394 ± 116 | 446 ± 227 |
| Vitamin B_12_ (μg) | 2.0 | 3.2 ± 2.4 | 2.7 ± 0.7 | 3.8 ± 3.3 |
| Vitamin C (mg) | 30 | 107 ± 116 | 118 ± 91 | 97 ± 139 |
| Vitamin E (mg) | 7.0 | 11.1 ± 6.1 | 11.5 ± 4.6 | 10.7 ± 7.5 |
|  |  |  |  |  |
| Caffeine (mg) | - | 178 ± 322 | 181 ± 349 | 174 ± 303 |
|  |  |  |  |  |

^1^Estimated average requirement (EAR) or adequate intake (AI) according to the Australia/New Zealand Nutrient Reference Values (1). The majority of recommendations are an EAR, except for fibre, potassium, sodium, and vitamin E which have an AI. Abbreviations: DFE, dietary folate equivalents.

## **Intervention compliance**

All participants met compliance requirements of consuming at least 80% of beverages. Compliance was excellent overall, with a range of 87.5 – 100% of beverages consumed and a total average of 97.2% and 95.5% of placebo and active beverages consumed, respectively. As expected, compliance declined across time for both intervention beverages with a greater variability in the number of drinks consumed by the final week of each intervention beverage (Supplementary Figure 1).

Reasons for not consuming all 7 drinks each week included: forgetting, illness, rescheduling their final follow-up appointment earlier (e.g. for work requirements), change in routine (e.g. travel for the weekend), or disliking the flavour. Nine participants reported changes to diet/lifestyle during the intervention period. Reasons include increase (n=4) or decrease (n=1) in exercise routine, a temporary shift in food/exercise due to illness (n=2), and a change to work routine/stress (n=2). Responses were discussed with participants at clinic visits, and none were considered a major diet or lifestyle change.


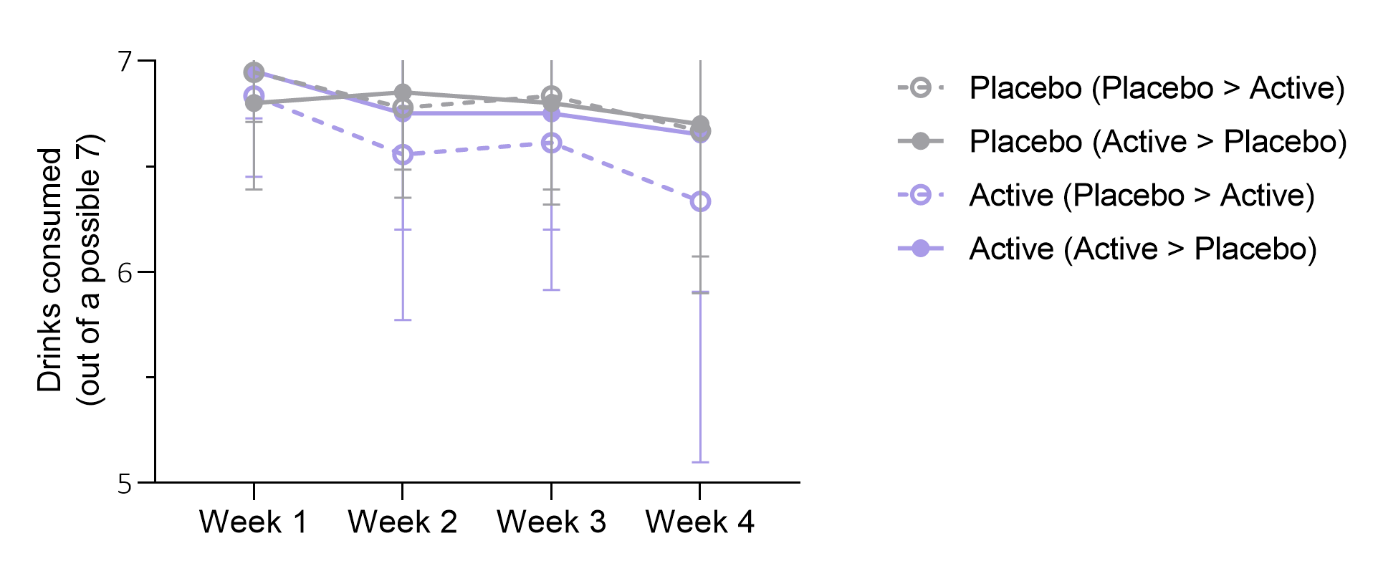


**Supplementary Figure 1. Compliance across the 4-week intervention period according to intervention group and sequence.**

## **Adverse events**

Participants were asked to record whether they noticed any side effects as a result of drinking their intervention beverages each week. Given that no severe adverse events were recorded, participants’ responses were cross-checked by researchers at monthly clinic visits. Gastrointestinal complaints were the most common adverse event reported for both active and placebo beverages, including both upper (reflux, belching, bloating, nausea) and lower (looser stools) gastrointestinal symptoms. Other adverse events included a dry cough, headaches, and a worsening of already existing eczema. Adverse events were predominantly mild, causing little to no stress to participants.

**Supplementary Table 2. Summary of adverse events reported for treatment beverages.**

| **Type of adverse event** | **Active intervention (n=38)** | **Placebo control (n=40)** |
| --- | --- | --- |
| Gastrointestinal |  |  |
| Mild | 4 (10%) | 7 (18%) |
| Moderate | 2 (5%) | 1 (3%) |
| Respiratory (mild) |  | 1 (3%) |
| Headaches (mild) | 1 (3%) |  |
| Skin (mild) |  | 1 (3%) |
